# GPs’ role security and therapeutic commitment in managing alcohol problems: a randomised controlled trial of a tailored improvement programme

**DOI:** 10.1186/1471-2296-15-70

**Published:** 2014-04-17

**Authors:** Myrna Keurhorst, Ivonne van Beurden, Peter Anderson, Maud Heinen, Reinier Akkermans, Michel Wensing, Miranda Laurant

**Affiliations:** 1 Radboud university medical center, Scientific Institute for Quality of Healthcare (IQ healthcare), P.O. Box 9101, 114 IQ healthcare, 6500 HB Nijmegen, The Netherlands; 2 Institute of Health and Society, Medical Faculty, Baddiley-Clark Building, Richardson Road, Newcastle NE2 4AX, UK; 3 HAN University of Applied Sciences, P.O. Box 6960, 6503 GL Nijmegen, The Netherlands

**Keywords:** Alcohol, Role security, Therapeutic commitment, Prevention, Primary healthcare, Implementation

## Abstract

**Background:**

General practitioners with more positive role security and therapeutic commitment towards patients with hazardous or harmful alcohol consumption are more involved and manage more alcohol-related problems than others. In this study we evaluated the effects of our tailored multi-faceted improvement implementation programme on GPs’ role security and therapeutic commitment and, in addition, which professional related factors influenced the impact of the implementation programme.

**Methods:**

In a cluster randomised controlled trial, 124 GPs from 82 Dutch general practices were randomised to either the intervention or control group. The tailored, multi-faceted programme included combined physician, organisation, and patient directed alcohol-specific implementation strategies to increase role security and therapeutic commitment in GPs. The control group was mailed the national guideline and patients received feedback letters. Questionnaires were completed before and 12 months after start of the programme. We performed linear multilevel regression analysis to evaluate effects of the implementation programme.

**Results:**

Participating GPs were predominantly male (63%) and had received very low levels of alcohol related education before start of the study (0.4 h). The programme increased therapeutic commitment (p = 0.005; 95%-CI 0.13 – 0.73) but not role security (p = 0.58; 95%-CI −0.31 – 0.54). How important GPs thought it was to improve their care for problematic alcohol consumption, and the GPs’ reported proportion of patients asked about alcohol consumption at baseline, contributed to the effect of the programme on therapeutic commitment.

**Conclusions:**

A tailored, multi-faceted programme aimed at improving GP management of patients with hazardous and harmful alcohol consumption improved GPs’ therapeutic commitment towards patients with alcohol-related problems, but failed to improve GPs’ role security. How important GPs thought it was to improve their care for problematic alcohol consumption, and the GPs’ reported proportion of patients asked about alcohol consumption at baseline, both increased the impact of the programme on therapeutic commitment. It might be worthwhile to monitor proceeding of role security and therapeutic commitment throughout the year after the implementation programme, to see whether the programme is effective on short term but faded out on the longer term.

**Trial registration:**

ClinicalTrials.gov Identifier: NCT00298220

## Background

Alcohol consumption is a leading determinant for the global burden of disease [1]. From 20 grams a day, the risk of harm accumulates: risk increases for chronic diseases, cancers, related neuropsychiatric conditions, intoxication, alcoholism, accidents, injuries and violence [2]. Furthermore, the costs related to alcohol are €125 billion a year in Europe for health, welfare, employment and criminal justice sectors as a consequence of alcohol-attributable disease, injury and violence [3]. In this study, safe to moderate consumption corresponds with 2–3 standard drinks per day for men and 1–2 for women, combined with 2 days per week without any consumption and maximum 5 and 3 drinks per occasion for men and women respectively. Hazardous and harmful alcohol consumption was assessed with the Alcohol Use Disorders Identification Test (AUDIT), an instrument developed for identification of these conditions. Scores 8–15 and 16–19 correspond with hazardous and harmful alcohol consumption, respectively [4].

Intervening in risky patterns of drinking through screening and brief interventions at an early stage is a cost-effective way to prevent drinking problems [5]. Primary healthcare is very suitable for such interventions because large numbers of patients with a wide range of consumption patterns can be reached [6-10]. Despite the evidence, such screening and brief interventions are rarely implemented into routine clinical practice [11,12]. Among the reasons most often cited are perceived lack of time, inadequate training, fear of antagonizing clients, the perceived incompatibility of brief alcohol intervention with primary healthcare, and the belief that those who are dependent on alcohol do not respond to interventions [11,13-15].

The engagement of general practitioners (GPs) in the prevention of alcohol problems can be explained by behavioural theories. The ASE model, which is based on the Theory of Planned Behaviour [16], is one of the models that often has been used to explain behaviours [17,18]. The model assumes that behaviour can be predicted by the behavioural intention, which is determined by the individual’s *A*ttitude, *S*ocial support and self-*E*fficacy. Moving from professional’s intentions to real actions depends on the person’s abilities and environmental barriers.

There is considerable evidence that GPs with more positive role security and therapeutic commitment towards patients with hazardous or harmful alcohol consumption are more involved and manage more alcohol-related problems than others [19-21]. Anderson et al. [20] have shown that GPs who received more education on alcohol; perceived that they were working in a supportive environment, expressed higher role security in working with alcohol problems, and reported greater therapeutic commitment to working with alcohol problems, were more likely to manage clients with alcohol-related problems. A negative attitude appeared to be an implementation barrier in behavioural change. Their training and support did not improve role security nor therapeutic commitment. The authors recommended that emotional responses of the GPs should be monitored more carefully in future quality improvement programmes, for example through on-site agents or facilitators [20]. Correspondingly, Funk et al. suggested to increase success rates of dissemination of brief interventions that support strategies that address therapeutic commitment, role security and beliefs more profoundly should be explored [22].

To further explore in what way GPs’ role security and therapeutic commitment could be positively influenced, we developed a tailored implementation programme, targeted at role security and therapeutic commitment and incorporated the above recommendations. This multi-faceted alcohol specific implementation strategy included professional, organisational and patient-directed strategies. We aimed to study 1) the effect of a multi-faceted implementation strategy on the providers’ role security and therapeutic commitment towards alcohol-related problems; and 2) other factors which can explain the changes in role security and therapeutic commitment towards alcohol-related problems.

## Methods

### Design and participants

Data used in this paper were part of the GPA-project (Engaging *G*eneral *P*ractice in the prevention of patients with *A*lcohol problems), a cluster randomised controlled trial [23] (trial number NCT00298220). This study assessed the effect of a tailored multi-faceted improvement programme on GPs’ screening of hazardous and harmful alcohol consumption and brief intervention rates as well as on role security and therapeutic commitment. Effects of this trial on screening and brief intervention rates and on patient reported alcohol consumption were described elsewhere [4,23].

Data were collected with measurements before (T0) and 12 months after (T1) delivery of the programme. In total, 2,758 Dutch general practices were invited during three recruitment waves. Practices could only enrol if all GPs in the practice agreed to participate. To encourage enrolment, the non-participants received a reminder after two weeks, and if necessary a second reminder after again two weeks. To encourage response at post measurement, we sent reminders after two and four weeks. Dependent on allocation group and the degree of participation in the different components of the programme, GPs were offered accreditation points, i.e. Permanent Education Points. Dutch clinicians – including GPs – are obliged to achieve sufficient accreditation points in order to maintain their medical license. Accreditation points could be achieved by educational activities and are ultimately granted by a department of the Royal Dutch Medical Association (KNMG).

The trial was approved by the Research Ethics Committee CMO of the region Arnhem-Nijmegen (letter dated 2 January 2006; SE/CMO 0003). The committee concluded in their letter that in compliance with the law on medical–scientific research (WMO), the GPA trial did not need approval. We asked for written informed consent, which was provided by all participants.

### Randomisation and allocation

The enrolled practices were randomised by a computerised scheme (block randomisation) to an equal-sized intervention group and control group. Randomisation was done at two moments: after the first two recruitment waves and again after the third wave. The improvement programme was offered to the general practices during October 2006 to June 2007 (intervention period). After randomisation, the practices were divided into clusters for logistic reasons, dependent on their location in the Netherlands. Clusters one through six (all from recruitment wave one or two) started the programme in October 2006 and their last possible activity was in May 2007. Clusters seven and eight (wave three) started the programme in December 2006 and also ended in May 2007. The programme was offered in eight clusters, but the content of those eight clusters was consistent. The research team organised and delivered the intervention, which made it impossible to blind the research team for practice allocation.

### Implementation programme

The intervention combined physician, organisation, and patient directed alcohol-specific implementation strategies. The emphasis was on educational training sessions and support visits by a trained facilitator, which were tailored to the providers’ needs and attitudes (see Table 1). The tailoring during training and during support visits was especially focused on the baseline role security and therapeutic commitment of the providers. During the first training session the baseline role security and therapeutic commitment of the providers were discussed and presumptions towards hazardous and harmful levels of alcohol consumption were addressed. Furthermore, the theoretical basics were discussed, i.e. definitions over risky alcohol consumption, epidemiology, risk of alcohol consumption, risk groups, symptoms and possible (brief) interventions. The second and third sessions focused on bringing theory into practice to overcome the barriers that hinder GPs. After a short summary of the theory about how to approach alcohol problems, the participants were able to revert to unfinished matters from the first session of support visit (if attended) or to bring in cases from their daily practice. Next, the GPs practiced motivational interviewing in role plays, a useful method in the treatment of lifestyle problems and disease. The focus in the role plays depended on the role security, therapeutic commitment and experiences of the participating GPs. During support visits barriers of the practice organisation as a whole, were addressed. First, remaining questions after the educational training sessions were discussed. Next, implementation barriers in daily practice were addressed. Besides practical tips to tackle structural, logistical and communication issues, the facilitator focused on the role security and therapeutic commitment of the practice team and discussed individual barriers to act upon alcohol problems. Staff delivering training and support strategies were trained using a detailed standardized protocol and written scripts and guidance.

**Table 1:** Outline of the intervention programme

GP directed interventions	
1	Distribution of the guideline on problematic alcohol consumption issued by the Dutch college of GPs.
2	A reminder-card to display on desk of the GP. This card featured the signs, symptoms and characteristics which should trigger a physician to ask about alcohol consumption. At the back site the Five Shot Test was listed, a five-item questionnaire to designed to estimate the amount of alcohol consumption of a patient, which is recommended in general practice because of its practical advantages and diagnostic properties.
3	Educational training session tailored to professionals’ attitudes. The entire general practice team (including practice assistants and nurses) was invited to participate in the small-scale training sessions (maximum around ten participants). Minimally one and maximally three sessions could be attended, tailored to the wishes, needs, and attitude of the teams. These sessions were offered to the practice teams in the early evening hours together with a light dinner (soup, bread, fruits). The duration of the sessions was between two and three hours. The basic content of the educational trainings was based on the guidelines of the Dutch college of GPs and on recent international guidelines. More in detail, the content was tailored to the attitudes of the GPs. In order to identify the attitudes towards and experiences with alcohol problems the Short Alcohol and Alcohol Problems Perception Questionnaire (SAAPPQ) was used. During the first training session the outcomes of the SAAPPQ were discussed and presumptions towards hazardous and harmful levels of alcohol consumption were addressed. Furthermore, the theoretical basics were discussed. And finally, the local addiction services were invited to participate in this session (see ‘*Organisation/practice directed interventions’*). The second and third sessions focussed on bringing theory into practice to overcome the barriers that hinder GPs. After a short summary of the theory about how to approach alcohol problems, the participants were able to revert to unfinished matters from the first session of support visit (if attended) or to bring in cases from their daily practice. Next, the GPs practiced motivational interviewing in role plays, a useful method in the treatment of lifestyle problems and disease. The casuistry in the role plays depended on the attitude and experiences of the participating GPs.
Organisation/practice directed interventions	
4	Feedback identifying the number of patients who are at risk because of their alcohol consumption. From the AUDIT patient questionnaires, distributed by the practice teams, the amount of alcohol consumption for each responding patient was calculated. The patients were divided into 4 categories: I. Safe to moderate drinker; II. Hazardous drinker; III. Harmful drinker; IV. Possibly dependant drinker. For each practice the proportion of patients in every category were calculated. The practices received this anonymous information together with the total number of returned patient questionnaires.
5	Facilitation of the cooperation with local addiction services for support and referral. The local addiction services were invited to join in the first educational training session. The goals were that the practice teams took cognizance of the experiences of the addiction services, that the GPs knew more precisely when to refer and what subsequently happened to their patients and to come to agreements about communication, accessibility, and cooperation.
6	Outreach visitor support by a trained facilitator tailored to needs of practice. Again, the entire practice team was invited and participation was tailored to the wishes and needs of the teams. Minimally one and maximally three support visits were offered. The visits took place during daytime and lasted around one hour. The content of the support visits was tailored to the barriers of the practice organisation as a whole. First, remaining questions after the educational training sessions were dealt with. Implementation barriers in daily practice were addressed next. Besides practical tips to tackle structural, logistical and communicative issues the facilitator focussed on the attitudes and beliefs of the practice team and discussed individual barriers to act upon alcohol problems.
Patient directed interventions	
7	Patient information letters about alcohol issued by the Dutch college of GPs and leaflets and self-help booklets issued by the NIGZ. These patient materials were offered to the general practices in order to be distributed by the GPs.
8	Poster in the waiting room. This gaudy poster drew the attention to alcohol with the advice to contact the GP or look at the websites of the NIGZ (National Institute for Health Promotion and Disease Prevention) or Trimbos (National institute of knowledge about mental healthcare, addiction services and societal care) for further information.
9	Personal feedback based on their alcohol consumption. The patients received a letter which cited the category to which they belonged and the corresponding advices. The advices were to turn to their GP or to look at the websites of the NIGZ or Trimbos. For patients in category I this was not necessary and for patients in category IV we added the advice to inquire at the local addiction service.

So, the intervention group received a partly standardised intervention, where education and support were tailored to individual needs. These parts of the programme were tailored as we hypothesised that GPs are more likely to increase their role security and therapeutic commitment in a tailored programme compared to a standardised programme which may not optimally match their baseline rates. Variability within the programme is inherent to tailoring and is expected to result in maximal improvement in role security and therapeutic commitment. Furthermore, physicians as well as patients received feedback from patient AUDIT scores [23], through personal feedback. For a detailed outline of the programme, see Table 1.

The control group was mailed the national guideline [24] (which was publicly available) and patient information letters on problematic alcohol consumption developed by the Dutch College of General Practitioners in order to distribute to patients by GPs when appropriate. The GPs did not receive further support or training. For ethical reasons, the patients also had to receive the personalised feedback on alcohol consumption in May 2007, which can be assessed as a minimal intervention, but took place after the physician programme ended.

### Outcome measurements

This paper describes outcomes on the GPs’ role security and therapeutic commitment. These were measured before and after the implementation programme, using the 10-item Shortened Alcohol and Alcohol Problems Perception Questionnaire (SAAPPQ). The SAAPPQ has been developed in England as a quick yet meaningful measure of GPs’ attitudes to working with drinkers, either as a way of measuring change over time or when planning intervention strategies [25]. We translated the questionnaire into Dutch, and independently back-translated it into English to check accuracy of the initial translation, both literally and idiomatically.

The role security domain within the SAAPPQ includes 2 sub-domains: role adequacy, and role legitimacy (e.g. “I feel I can appropriately advise my patients about drinking and its effects”; “I feel I have the right to ask patients questions about their drinking when necessary”). Therapeutic commitment involves motivation, task specific self-esteem, and work satisfaction. Within the scales of role security and therapeutic commitment (ratings on a 7-point Likert scale ranging from ‘strongly agree’ to ‘strongly disagree’) means were calculated. For the questions of the SAAPPQ with additional scoring key, see Additional file 1.

Additionally, we added questions to the pre-measurement questionnaire about the providers’ characteristics, such as age, gender, practice size (number of patients per physicians), full-time equivalent (FTE), and the degree of urbanisation of the practice. Moreover, we asked how important GPs thought it was to improve their identification of patients with alcohol-related problems (both before and after intervention), how important GPs thought it was to improve care for patients with alcohol-related problems (both before and after intervention), degree of alcohol-related education, the GPs’ reported proportion of patients asked about their alcohol consumption (both before and after intervention), proportion of patients counselled by the GP for alcohol-related problems (both before and after intervention), degree of participation in the intervention programme, and correct or incorrect estimating the maximum number of drinks by the guideline (both before and after intervention). The post measurement questionnaire was similar although questions about provider characteristics such as practice type, patient load, etc. were excluded.

### Sample size

A power calculation was carried out to estimate the number of practices to be included to detect the effect of the implementation programme in changing providers’ advice giving behaviour and is described elsewhere [23]. We intended to recruit 80 general practices.

### Statistical analysis

Practice was the unit of allocation. Because of the hierarchical structure (GPs nested within practices), we performed a linear multilevel (mixed model) analysis. In this analyses we take account of the variability associated with each level of nesting. In a mixed model both fixed and random effects can be analysed. We performed a model with a random intercept for practices and all other variables fixed, as these were used to correct the effect. Subsequently, we investigated the effect of the implementation programme on domains of role security and therapeutic commitment as a continuous outcome variable. Multilevel linear regression analysis with the follow up score as outcome and baseline score as covariate was used to evaluate the effect of the implementation programme. Descriptive statistics were used to describe the characteristics of participating GPs at baseline.

During the second wave, the SAAPPQ had a systematic flaw. The last SAAPPQ-question “In general, I like drinkers” was systematically missing and caused a missing value, concerning 67 GPs. All participants that had missings in their measurements, were assigned a value based on multiple imputation procedure. All of the potential determinants of effects were used for calculating the imputation. It is suggested that multiple imputation yields less bias and less variability than the often used last observation carried forward method [26]. Before the multiple imputation, we checked all the variables in the absence of a significant difference between the group with the systematic missing (i.e. second wave), to the group without the significant missing (i.e. first and third wave). After multiple imputation, the sample did not significantly differ from the former sample without multiple imputation. To maintain the power, we decided to proceed with the multiple imputation sample.

We added the following factors from baseline separately to explain the changes in the effects on role security and therapeutic commitment towards alcohol-related problems, as we thought they might determine the effect of the implementation programme: age, gender, full time equivalent, size of patient population, working area, practice setting (solo, duo, group, etc.), how important GPs thought it was to improve their identification of patients with alcohol-related problems (both before and after intervention), how important GPs thought it was to improve care for patients with alcohol-related problems (both before and after intervention), degree of alcohol-related education, the GPs’ reported proportion of patients asked about their alcohol consumption (both before and after intervention), proportion of patients counselled by the GP for alcohol-related problems (both before and after intervention), degree of participation in the intervention programme, and correct or incorrect estimating the maximum number of drinks by the guideline (both before and after intervention). Furthermore, we added interaction terms in order to identify interactive effect of the programme (effect modification)*.* We considered a p-value < 0.05 statistically significant. Descriptive analyses was conducted using SPSS version 20.0 (IBM PASW statistics 20) and multilevel regression analyses was conducted using SAS V9.2 (SAS Institute Inc., Cary, NC, USA).

## Results

### Study population

**Figure 1:**
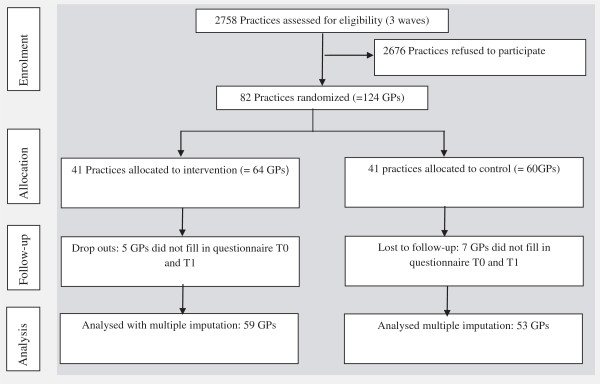
Participant flow.

Figure 1 outlines the study design and the flow of participating practices and GPs. The participating 82 practices with 124 GPs were randomised. After randomisation but before pre measurement, five practices withdrew: one in the intervention group and four in the control group (no data available). This resulted in 40 practices (63 GPs) receiving allocated intervention and 37 practices (56 GPs) in the control group.

With regard to the baseline and follow-up measurement, 112 GPs delivered sufficient information to be included in the analysis: 59 in the intervention group and 53 in the control group.

Descriptive demographics of the GPs are detailed in Table 2. GPs of the intervention and control group only significantly differed in age: GPs of the intervention group turned out to be on average 4 years younger than their colleagues in the control group, but in both groups the majority of GPs was middle-aged (45–50 years).

**Table 2:** Baseline characteristics of participating and non-participating GPs

***Characteristic***	***Intervention (N = 59)***	***Control (N = 53)***	***Non-participants (N = 761)***
Male gender	39 (66%)	31 (58.5%)	478 (65.4%)^#^
Mean age at start of study (SD)	45 (6.9)^#^	49 (7.7)^#^	48.1 (8.0)
Mean FTE (SD)	0.84 (0.2)	0.97 (1.2)	0.83 (0.56)
Mean size of patient population (SD)	2158 (627)	2179 (730)	2153 (689)
*Working area*			
Rural	18 (31%)	14 (26%)	148 (20%)
Urbanised rural	23 (39%)	16 (57%)	306 (41%)
Urban	3 (5%)	10 (19%)	142 (19%)
Big city	15 (25%)	13 (25%)	145 (20%)
*Practice type*			
Solo	20 (34%)	24 (45%)	193 (26%)^#^
Duo	23 (39%)	22 (42%)	216 (29%)^#^
Group	10 (17%)	5 (9%)	213 (29%)^#^
Health Centre	6 (10%)	2 (4%)	79 (11%)^#^
Other			42 (6%)^#^
Average hours of training in alcohol problems before start of study (SD)	0.51 (1.1)	0.36 (0.97)	n.m.
*Role security*^*¥*^: total (SD)*	*5.0 (0.91)*	*5.1 (0.76)*	*5.2 (0.82)*
Role adequacy	4.4 (1.06)	4.4 (1.12)	4.6 (1.05)
Role legitimacy	5.6 (1.20)	5.7 (1.04)	5.7 (1.07)
*Therapeutic commitment*^*±*^: total (SD)*	*3.9 (0.92)*	*3.9 (0.74)*	*3.9 (0.76)*
Task-specific self-esteem	3.9 (1.14)	3.7 (1.22)	4.0 (1.11)
Work satisfaction	3.3 (1.32)	3.5 (1.20)	3.6 (0.88)
Motivation	4.5 (1.06)	4.5 (1.01)	4.2 (1.10)

^#^Significant difference (p < 0.05) compared to participating GPs; n.m. = not measured; ^*¥*^*=* Role security is calculated by the average of role adequacy and role legitimacy; ^*±*^Therapeutic commitment is calculated by the average of task-specific self-esteem, work satisfaction and motivation; *minimal role security = 1 and maximum role security = 7.

Table 2 also includes baseline role security and therapeutic commitment. The role security and therapeutic commitment levels of the control group are not different compared to the intervention group. For results on the 10 single SAAPPQ questions from which role security and therapeutic commitment in Table 2 were calculated, see Additional file 2.

### Non-participants

The non-participant questionnaire was returned by 761 GPs (28%). As to age, average percentage of fulltime work, caseload, and working area the participating GPs did not differ from the non-participants (see Table 2). However, the non-participating GPs worked on average in practices with more colleagues than the participating GPs, who mostly worked in solo- or duo practices (p < 0.05). Moreover, the non-participant population consisted of more male GPs compared to the participating population, but just with a 2% difference (p < 0.05). Non-participant role security and therapeutic commitment did not differ from participant baseline levels. For results on the 10 single SAAPPQ questions from which role security and therapeutic commitment were calculated, see Additional file 2.

### Changes in role security and therapeutic commitment

Table 3 shows scores before and after the implementation of the programme, and the mean difference for role security and therapeutic commitment, respectively. GPs in both intervention and control groups improved in their role security and therapeutic commitment, though the intervention group showed a higher mean score.

**Table 3:** Role security and therapeutic commitment before and after intervention

***Role security***			
*Allocation*	*Mean before (SD)*	*Mean after (SD)*	*Mean difference (SD)*
Intervention group	5.01 (0.91)	5.58 (0.79)	0.59 (1.11)
Control	5.08 (0.76)	5.46 (0.61)	0.31 (0.72)
***Therapeutic commitment***			
*Allocation*	*Mean before (SD)*	*Mean after (SD)*	*Mean difference (SD)*
Intervention group	3.92 (0.92)	4.58 (0.81)	0.63 (0.97)
Control	3.90 (0.74)	4.02 (0.76)	0.20 (0.64)

Corrected for baseline levels, Table 4 shows the results of the multilevel analysis without and with multiple imputation for both role security and therapeutic commitment respectively. From this table we can see there were no major changes in parameters, which allows us to proceed with the multiple imputation model. The multilevel regression analysis showed that GPs in the intervention group improved in their therapeutic commitment more than 0.43 on the 7-point likert scale (95%-CI 0.13-0.73) compared to GPs in the control group. On the contrary, role security did not significantly change due to the intervention (β = 0.11; p = 0.58; 95%-CI −0.31-0.54).

**Table 4:** Role security and therapeutic commitment with and without multiple imputation

***Role security***				
*Allocation*	β*	*95%-CI*	*S.E.*	*p-value*
Intervention effect without multiple imputation	0.13	−0.18 – 0.44	0.16	0.4111
Intervention effect with multiple imputation	0.12	−0.31 – 0.54	0.21	0.5791
***Therapeutic commitment***				
*Allocation*	β*	*95%-CI*	*S.E.*	*p-value*
Intervention effect without multiple imputation	0.52	0.21 – 0.83	0.16	0.0017
Intervention effect with multiple imputation	0.43	0.13 – 0.73	0.15	0.0052

*Improvement on 7-point likert scale; 95%-CI = 95% Confidence Interval; S.E. = Standard Error.

### Explaining changes in role security and therapeutic commitment change

With regard to therapeutic commitment, how important GPs thought it was to improve their care for problematic alcohol consumption, and the GPs’ reported proportion of patients asked about alcohol consumption at baseline, were identified as likely determinants of effects (p < 0.15). The results from Table 5 show that, corrected for these both factors, the intervention effect further increased compared to the uncorrected intervention effect from 0.43 to 0.51 points within therapeutic commitment.

**Table 5:** Determinants of intervention effect on role security and therapeutic commitment

***Role security***				
*Allocation*	β*	*95%-CI*	*S.E.*	*p-value*
Intervention effect	−0.17	−0.85 – 0.51	0.32	0.6029
Pre measurement	0.16	−0.07 – 0.38	0.11	0.1697
Participation degree	0.42	−0.11 – 0.96	0.26	0.1144
***Therapeutic commitment***				
*Allocation*	β*	*95%-CI*	*S.E.*	*p-value*
Intervention effect	0.51	0.23 – 0.80	0.14	0.0006
Pre measurement	0.60	0.32 – 0.89	0.13	0.0008
GP reported importance to improve care	0.16	0.05 – 0.28	0.06	0.0079
Proportion of patients of which the GP asked for their alcohol consumption	0.01	−0.00 – 0.02	0.01	0.0654

*Improvement on 7-point likert scale; 95%-CI = 95% Confidence Interval; S.E. = Standard Error.

Role security did not improve due to the implementation programme. The degree of participation in the intervention programme was identified as a likely determinant of effect (p < 0.15), but the intervention effects remained to be non-significant. This implies that the programme did not affected role security neither in a negatively, nor positively.

Furthermore, we were not able to identify subgroups in intervention effects in terms of effect modification. Neither in the effect of the implementation programme on role security, nor on therapeutic commitment, interactive effects between potential determinants of effect could be identified.

## Discussion

The main finding of this study was that our implementation programme improved the GPs’ therapeutic commitment, but despite all efforts for tailoring the intervention to the providers, it did not affect the role security. In line with this latter finding, the tailored implementation programme neither improved GPs’ screening and brief intervention rates, as described elsewhere [23].

How important GPs thought it was to improve their care for problematic alcohol consumption, and the GPs’ reported proportion of patients asked about alcohol consumption at baseline, were identified as determinants of effects on the therapeutic commitment. Nevertheless, when corrected for this, the programme remained to be effective in improving therapeutic commitment. With regard to role security, no determinants of effects were identified.

Our findings do not confirm our hypothesis that our tailored implementation programme would improve GPs’ role security, despite the efforts for tailoring to providers’ baseline role security. Role security is about having knowledge and skills in recognising and discussing risky alcohol consumption and role legitimacy. Looking at the professional-oriented elements of the programme that could have influenced role security, the educational sessions and support visits were tailored to the GPs’ SAAPPQ baseline scores in the sense that the outcomes of the SAAPPQ were discussed and presumptions towards hazardous and harmful levels of alcohol consumption were addressed. A possible explanation for improvement of therapeutic commitment and not for role security might be the fact that the baseline level of role security was higher than the level of therapeutic commitment, and was therefore leaving less room for improvement. Another reason for not showing a substantial improvement of role security might be that follow-up was measured only after 12 months. An initial effect on role security might have faded out by that time. Additionally, in line with our finding that the programme did not increase role security, was the fact that the study results did not show improved screening and brief intervention rates. The screening and brief intervention rates were initially improved during the implementation period, but the effects deteriorated at the end, i.e. there was no difference between the intervention group and control group [23]. This may imply that only if both role security and therapeutic commitment are improved this will have an impact on provider behaviour. Furthermore, when looking at the two sub-domains within role security, one could expect it to be easier to improve role adequacy (knowledge and skills) than role legitimacy (Additional file 1). The results indeed showed a larger improvement in role adequacy than in role legitimacy, however the difference was not sufficient to draw any firm conclusions from. It might be indicating however that more attention needs to be given to enhancing role legitimacy.

Also, similar to our findings, Anderson et al. found that training and support did not increase role security. In fact, they even found that role security and therapeutic commitment for GPs who were already role-insecure and low therapeutically committed, actually deteriorated [20]. We cannot confirm this finding that experience in screening and brief interventions deteriorates role security in GPs who were already insecure in their role, though we saw that how important GPs thought it was to improve their care for problematic alcohol consumption, and the GPs’ reported proportion of patients asked about alcohol consumption at baseline, facilitated improvement of therapeutic commitment. Tailoring the intervention to the GPs’ levels of role security, as we did, however, might not be sufficient to actually improve role security and subsequently screening and brief intervention behaviour. In the study of Butler et al. [27], it was emphasised to not just tell GPs to incorporate behaviour change counselling into their consults, but training requires more finesse in the sense that perceptions and the internally-driven processes of GPs are addressed in the training and support sessions. We think our study incorporated those elements, this may explain why we succeeded in improving therapeutic commitment.

Although the participants were only a small proportion of the total population, they largely reflected key characteristics of GPs in The Netherlands. Only on the aspect of gender and on practice type, there was just a small difference between participating and non-participating GPs. This means that it is very likely that our results are representative for the Dutch GP population. This is interesting, since the recruitment of practices was laborious and we had to invite more practices than anticipated. Experiences from colleagues in international clinical trials learn us that it is increasingly difficult to recruit and retain GPs for clinical trials [28-31].

On the other side, a limitation of our study was the very limited degree of participation of the GPs in the training sessions and visits: only 59% of GPs from the intervention group met the minimal demands of enrolment. This possibly means that the acceptability of the implementation programme was suboptimal. Furthermore, we were not able to identify different effects of the implementation programme between subgroups of patients (i.e. effect modification). It is likely that the sample size was too small to detect possible effect modification. Besides, during a systematic flaw of missing a question in a part of the questionnaires, we required multiple imputation to maintain as much power as possible. However, after checking whether multiple imputation affected the results, there were no signs of any affected results.

Although we have shown that it is possible to improve GPs’ therapeutic commitment, it was described in an earlier published article of this study that the implementation programme neither produced improved screening and brief intervention outcomes at the GP level [23], nor on the level of patient alcohol consumption [4] both at one year follow-up. Like suggested in an earlier article of this GPA-project [23], this does not necessarily mean that the implementation programme did not work, as the transtheoretical model of (health) behaviour change suggests that it can take up to five years for new behaviour to be integrated in daily routines [32]. Also, as researchers we might be too keen on having effects from implementation strategies, which results in high expectations and ambitious, high intensity implementation programmes. Probably it is more effective to take very small steps in the process of GPs incorporating prevention activities, since their practice actually is more focused on the disease model. That means that we should think about other strategies to increase role security and therapeutic commitment, find out the optimal measurement times and frequencies, and create long-term trials to monitor role security, therapeutic commitment and in the end screening and brief interventions against hazardous and harmful alcohol consumption.

Furthermore, research implementation programmes could focus on letting the implementation strategy for screening and brief interventions match as much as possible to GPs’ current practice in a way of achieving ‘personalised implementation’ , which likely is to be focused on the disease model. In addition, if it remained to be difficult to improve GPs’ readiness to screen and do brief interventions, one might not use (solely) professional oriented implementation strategies aimed at GPs, but on the contrary test the effect of organisational oriented implementation strategies like physically locating addiction care in the general practice or test the effect of substitution of preventive tasks from the GP to practice nurses. The latter might be a more low-threshold intervention. There are studies that already evaluated the effects of nurses’ SBI (e.g. [33-35]), this research could be extended with evaluating task substitution from the GP to the nurse.

Lastly, it would be worthwhile to gain more insight of the GPs’ attitudes over time. We had a long time between the first and last measurement, which resulted in a kind of ‘black box’ with regard to the attitude in due course. If it was shown that the effects faded out in time, a short booster programme may be effective in maintaining improved role security and therapeutic commitment, and maybe even maintaining improved screening and advice giving behaviour.

## Conclusions

A tailored, multi-faceted programme aimed at improving GP management of patients with hazardous and harmful alcohol consumption improved GPs’ therapeutic commitment towards patients with alcohol-related problems, but failed to improve GPs’ role security. How important GPs thought it was to improve their care for problematic alcohol consumption, and the GPs’ reported proportion of patients asked about alcohol consumption at baseline contributed to the effect of the programme on therapeutic commitment. It might be worthwhile to monitor proceeding of role security and therapeutic commitment throughout the year after the implementation programme, to see whether the programme is effective on short term but faded out.

## Abbreviations

GP: General practitioner; GPA: Engaging General Practice in the prevention of patients with Alcohol problems; SAAPPQ: Shortened Alcohol and Alcohol Problems Perception Questionnaire.

## Competing interests

The authors declare that they have no competing interests.

## Authors’ contributions

ML, PA and MW were responsible for designing the study protocol and set up of the implementation programme. IvB conducted the study. MK and RA analysed the data and interpreted the results. MK drafted the first manuscript, critically revised the draft manuscripts. ML and MH assisted with the in interpretation of the results and the drafting the manuscripts. All authors have read and approved the final manuscript.

## Pre-publication history

The pre-publication history for this paper can be accessed here:

http://www.biomedcentral.com/1471-2296/15/70/prepub

## Supplementary Material

Additional file 1 **The SAAPPQ questionnaire (English version) with scoring key.** This file shows the 10 single SAAPPQ questions and includes the scoring key for calculating role security and therapeutic commitment. Click here for file

Additional file 2 **Baseline role security and therapeutic commitment of participating and non-participating GPs.** This file shows baseline results from the 10 single SAAPPQ questions, from which the Table 2 role security and therapeutic commitment were calculated. Click here for file
