# Supplementary material for: GPs’ role security and therapeutic commitment in managing alcohol problems: a randomised controlled trial of a tailored improvement programme
Source: BMC Fam Pract. 2014 Apr 17;15:70. doi: 10.1186/1471-2296-15-70 (PMC4021502; doi:10.1186/1471-2296-15-70)
Supplement: Additional file 1 — The SAAPPQ questionnaire (English version) with scoring key. This file shows the 10 single SAAPPQ questions and includes the scoring key for calculating role security and therapeutic commitment. [file 1471-2296-15-70-S1.docx]

**Additional file 1: The SAAPPQ questionnaire (English version) with scoring key**

|  |  | **Strongly agree** | **Quite strongly agree** | **Agree** | **Neither agree or disagree** | **Disagree** | **Quite strongly disagree** | **Strongly disagree** |
| --- | --- | --- | --- | --- | --- | --- | --- | --- |
|  |  | 1 | 2 | 3 | 4 | 5 | 6 | 7 |
| 1 | I feel I know enough about causes of drinking problems to carry out my role when working with drinkers |  |  |  |  |  |  |  |
| 2 | I feel I can appropriately advise my patients about drinking and its effects |  |  |  |  |  |  |  |
| 3 | I feel I do not have much to be proud of when working with drinkers |  |  |  |  |  |  |  |
| 4 | All in all I am inclined to feel I am a failure with drinkers |  |  |  |  |  |  |  |
| 5 | I want to work with drinkers |  |  |  |  |  |  |  |
| 6 | Pessimism is the most realistic attitude to take towards drinkers |  |  |  |  |  |  |  |
| 7 | I feel I have the right to ask patients questions about their drinking when necessary |  |  |  |  |  |  |  |
| 8 | I feel that my patients believe I have the right to ask them questions about drinking when necessary |  |  |  |  |  |  |  |
| 9 | In general, it is rewarding to work with drinkers |  |  |  |  |  |  |  |
| 10 | In general I like drinkers |  |  |  |  |  |  |  |

**Scoring**

*Reverse scoring for items 1, 2, 5, 7, 8, 9, 10*

*Role adequacy: Add scores on items 1, 2*

*Role legitimacy: Add scores on items 7, 8*

*Motivation: Add scores on items 5, 6*

*Task-specific self-esteem: Add scores on items 3, 4*

*Work satisfaction: Add scores on items 9, 10*

*Role security: Add scores on role adequacy and role legitimacy*

*Therapeutic commitment: Add scores on Motivation, Work Satisfaction and Task-specific Self-esteem*
