# Supplementary material for: GPs’ role security and therapeutic commitment in managing alcohol problems: a randomised controlled trial of a tailored improvement programme
Source: BMC Fam Pract. 2014 Apr 17;15:70. doi: 10.1186/1471-2296-15-70 (PMC4021502; doi:10.1186/1471-2296-15-70)
Supplement: Additional file 2 — Baseline role security and therapeutic commitment of participating and non-participating GPs. This file shows baseline results from the 10 single SAAPPQ questions, from which the Table 2 role security and therapeutic commitment were calculated. [file 1471-2296-15-70-S2.docx]

**Additional file 2: Baseline role security and therapeutic commitment of participating and non-participating GPs**

|  |  | *Intervention (N=59)** | *Control (N=53)** | *Non-participants (N=761)** |
| --- | --- | --- | --- | --- |
| 1 | I feel I know enough about causes of drinking problems to carry out my role when working with drinkers (mean; SD) | 3.9 (1.40) | 3.8 (1.3) | 3.6 (1.22) |
| 2 | I feel I can appropriately advise my patients about drinking and its effects (mean; SD) | 3.3 (1.18) | 3.4 (1.30) | 3.2 (1.15) |
| 3 | I feel I do not have much to be proud of when working with drinkers (mean; SD) | 3.9 (1.31) | 3.8 (1.54) | 3.9 (1.32) |
| 4 | All in all I am inclined to feel I am a failure with drinkers (mean; SD) | 3.8 (1.27) | 3.5 (1.31) | 4.0 (1.32) |
| 5 | I want to work with drinkers (mean; SD) | 3.6 (1.14) | 3.6 (1.34) | 4.2 (1.33) |
| 6 | Pessimism is the most realistic attitude to take towards drinkers (mean; SD) | 4.6 (1.39) | 4.6 (1.54) | 4.6 (1.47) |
| 7 | I feel I have the right to ask patients questions about their drinking when necessary (mean; SD) | 2.0 (1.36) | 1.9 (1.23) | 1.9 (1.18) |
| 8 | I feel that my patients believe I have the right to ask them questions about drinking when necessary (mean; SD) | 2.7 (1.40) | 2.6 (1.30) | 2.6 (1.24) |
| 9 | In general, it is rewarding to work with drinkers (mean; SD) | 4.8 (1.42) | 4.8 (1.31) | 4.9 (1.20) |
| 10 | In general I like drinkers (mean; SD) | 4.3 (1.08) | 3.4 (1.14) | 3.9 (1.05) |

* Scores on 7-point likert scale, in which 1= Strongly agree to 7=Strongly disagree
